# Gene model for the ortholog of *Mipp2* in *Drosophila simulans*

**DOI:** 10.17912/micropub.biology.000765

**Published:** 2025-09-14

**Authors:** Graham M. Jones, Natalie Ball, Alejandra Moreno, Cody Reece Puckett, Cindy Wolfe, Claudia Uhde-Stone, Nikolaos Tsotakos, Geoffrey D. Findlay, Chinmay P. Rele, Laura K Reed

**Affiliations:** 1 The University of Alabama, Tuscaloosa, AL USA; 2 California State University, East Bay, Hayward, CA US; 3 Kentucky Wesleyan College, Owensboro, KY USA; 4 Penn State Harrisburg, Middletown, PA USA; 5 College of the Holy Cross, Worcester, MA USA

## Abstract

Presenting a gene model for the ortholog of multiple inositol polyphosphate phosphatase 2 ( *Mipp2* ) in the May 2017 (Princeton ASM75419v2/DsimGB2) Genome Assembly (GenBank Accession: GCA_000754195.3 ) of *Drosophila simulans* . This ortholog was characterized as part of a developing dataset to study the evolution of the Insulin/insulin-like growth factor signaling pathway (IIS) across the genus *Drosophila* using the Genomics Education Partnership gene annotation protocol for Course-based Undergraduate Research Experiences.

**Figure 1. Genomic neighborhood and gene model for Mipp2 in D. simulans:**
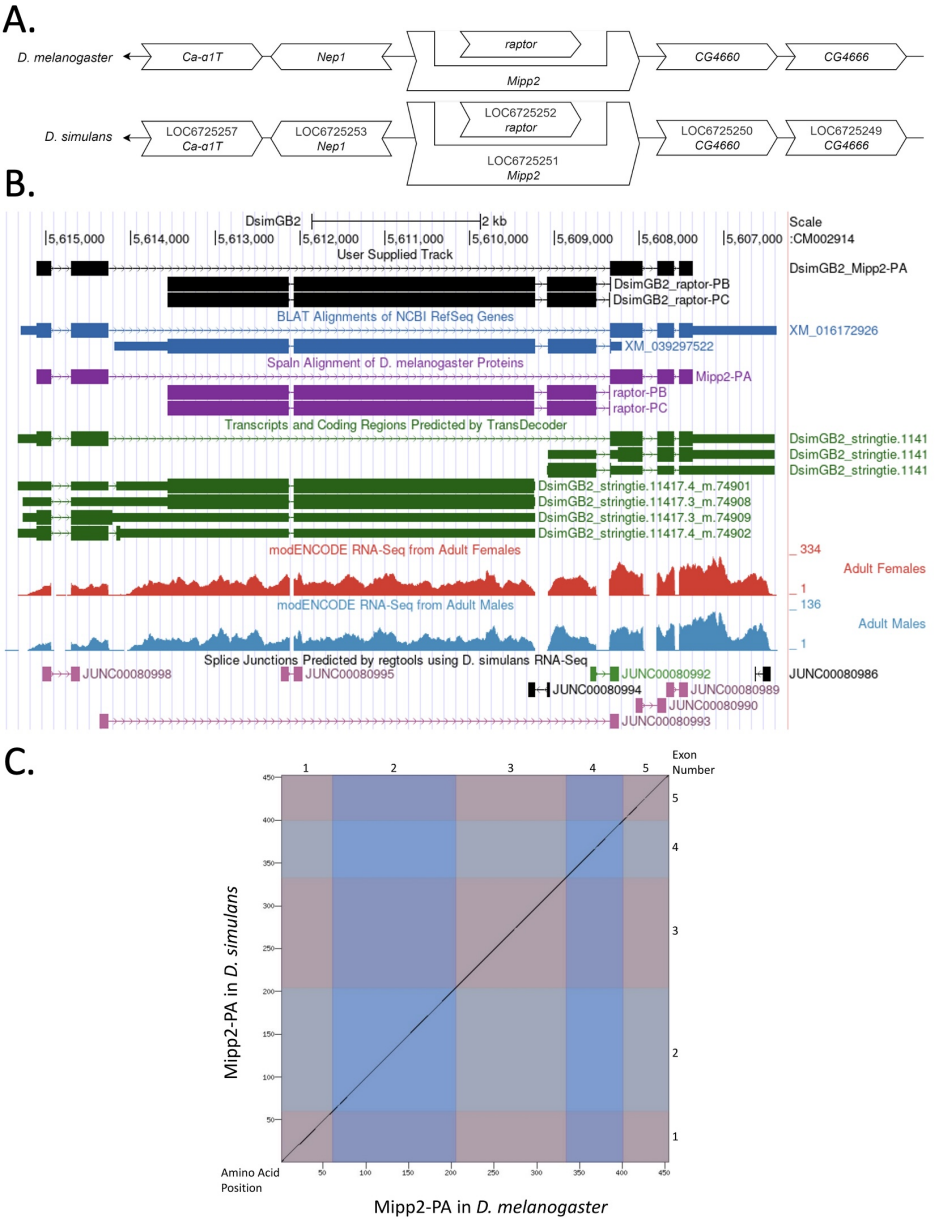
(A) Synteny of genomic neighborhood of *Mipp2* in *D. melanogaster* and *D. simulans* . Gene arrows pointing in the same direction as target gene in both *D. simulans* and *D. melanogaster* are on the same strand as the target gene; gene arrows pointing in the opposite direction are on the opposite strand. The thin black underlying arrow pointing to the left indicates that *Mipp2* is on the minus (–) strand in both species. White arrows in *D. simulans* indicate the locus ID and the orthology to the corresponding gene in *D. melanogaster* . The locus identifiers are specific to *D. simulans* . (B) Gene Model in UCSC Track Hub (Raney et al. 2014): the gene model in *D. simulans* (black), Spaln of *D. melanogaster* proteins (purple, alignment of refseq proteins from *D. melanogaster* ), BLAT alignments of NCBI RefSeq Genes (blue, alignment of refseq genes for *D. simulans* ), RNA-Seq from adult females and males (red and blue respectively, alignment of Illumina RNA-Seq reads from *D. simulans* ), and Transcripts (green) including coding regions predicted by TransDecoder and Splice Junctions Predicted by regtools using *D. simulans* RNA-Seq (Gravely et al. 2010; SRP006203 ). Splice junctions shown have a minimum read-depth of 102 with 100-499 supporting reads in pink. The custom gene model (User Supplied Track) is indicated in black with CDS depicted with wide boxes, intron with narrow lines (arrows indicate direction of transcription). (C) Dot Plot of Mipp2-PA in *D. melanogaster* ( *x* -axis) vs. the orthologous peptide in *D. simulans* ( *y* -axis). Amino acid number is indicated along the left and bottom; CDS number is indicated along the top and right, and CDSs are also highlighted with alternating colors.

## Description

**Table:** 

*This article reports a predicted gene model generated by undergraduate work using a structured gene model annotation protocol defined by the Genomics Education Partnership (GEP; thegep.org) for Course-based Undergraduate Research Experience (CURE). The following information in this box may be repeated in other articles submitted by participants using the same GEP CURE protocol for annotating Drosophila species orthologs of Drosophila melanogaster genes in the insulin signaling pathway.* "In this GEP CURE protocol students use web-based tools to manually annotate genes in non-model *Drosophila* species based on orthology to genes in the well-annotated model organism fruitfly *Drosophila melanogaster* . The GEP uses web-based tools to allow undergraduates to participate in course-based research by generating manual annotations of genes in non-model species (Rele et al., 2023). Computational-based gene predictions in any organism are often improved by careful manual annotation and curation, allowing for more accurate analyses of gene and genome evolution (Mudge and Harrow 2016; Tello-Ruiz et al., 2019). These models of orthologous genes across species, such as the one presented here, then provide a reliable basis for further evolutionary genomic analyses when made available to the scientific community.” (Myers et al., 2024). “The particular gene ortholog described here was characterized as part of a developing dataset to study the evolution of the Insulin/insulin-like growth factor signaling pathway (IIS) across the genus *Drosophila* . The Insulin/insulin-like growth factor signaling pathway (IIS) is a highly conserved signaling pathway in animals and is central to mediating organismal responses to nutrients (Hietakangas and Cohen 2009; Grewal 2009).” (Myers et al., 2024). “ *D. simulans* (NCBI:txid7240) is part of the *melanogaster* species group within the subgenus *Sophophora* of the genus *Drosophila* (Sturtevant 1939; Bock and Wheeler 1972) *.* It was first described by Sturtevant (1919). *D. simulans* is a sibling species to *D. melanogaster* , thus extensively studied in the context of speciation genetics and evolutionary ecology (Powell 1990). Historically, *D. simulans* was a tropical species native to sub-Saharan Africa (Lemeunier et al., 1986) where figs served as a primary host (Lachaise and Tsacas 1983). However, *D. simulans's* range has expanded worldwide within the last century as a human commensal using a broad range of rotting fruits as breeding sites (https://www.taxodros.uzh.ch, accessed 1 Feb 2023).” (Lawson et al., 2024).

The model presented here is the ortholog of *Mipp2* in the DsimGB2 assembly of *D. simulans* (Drosophila 12 Genomes Consortium et al. 2007; GCA_000754195.3 ) and is othologous to the Gnomon Peptide ID ( XP_016038261.1 ) predicted model in *D. simulans* ( LOC6725251 ) *.* This gene model is based on RNA-Seq data from *D. simulans* (Gravely et al. 2010; SRP006203 *)* and the *Mipp2* ( GCA_000001215.4 ) in *D. melanogaster* from FB2022_02 (Larkin et al., 2021, Gramates et al., 2022; Jenkins et al., 2022).

*Mipp2* , or *multiple inositol polyphosphate phosphatase 2* , is an ortholog of human *Minpp1* (provided by Alliance) and is predicted to have or enable acid phosphatase, bisphosphogylcerate 3-phosphatase and inositol phosphate phosphatase activities (String Consortium 2022). The Mipp2 protein is predicted to be part of the TORC1 complex by interacting with TSC1 (Housden et al., 2015). Importantly, *Mipp2* has been identified as a cis eQTL in a multiparent *Drosophila* population in multiple nutrient environments, including control and high-sugar diets (Stanley et al., 2017). RNAi knockdown of *Mipp2* in *Drosophila* S2 cells caused reduced cell size and an increased fraction of cells in the G1 phase of the cell cycle (Bjorklund et al., 2006). At least one isoform of the human ortholog, *Minpp1* , is secreted into extracellular exosomes (Zubair et al., 2022).

***Synteny***

*Mipp2* is located on the X chromosome in *D. melanogaster* and is flanked by *Ca-alpha1T* and *Nep1* upstream and *CG4660* and *CG4666* downstream. *Mipp2* nests *raptor .* We determined that the putative ortholog of *Mipp2* is found on scaffold CM002914 in *D. simulans* with LOCUS ID LOC6725251 (via *tblastn* search with an e-value of 0.0 and percent identity of 95.82%). The LOCUS IDs of the surrounding genes are LOC6725257 , LOC6725253 , LOC6725252 , LOC6725250 , LOC6725249 which are othologous to *Ca-alpha1T , Nep1 , raptor , CG4660 , and CG4666* in *D. melanogaster* with percent identities 97.42%, 98.82%, 97.56%, 97.70%, and 100% and e-values of 0.0, 0.0, 0.0, 0.0, and 6e-145 respectively, as determined by *blastp* ( Figure 1A, Altschul et al., 1990). We believe this is the correct ortholog assignment for *Mipp2* in *D. simulans* because all downstream, upstream, and nested genes in the genomic neighborhood of *Mipp2* in *D. simulans* have a very high percent identity with genes in the same relative locations in *D. melanogaster.*

***Protein Model***

*Mipp2* in *D. melanogaster* has one protein coding isoform (Mipp2-PA), and similarly, based on sequence conservation and parsimony, we find evidence for only one protein coding isoform in *D. simulans* ( Figure 1B ). Isoform Mipp2-PA contains five CDSs. Note that the entire gene length of *raptor* nests within the second intron of *Mipp2* , this results in additional splice sites mapping within the gene span of *Mipp2* that do not belong to *Mipp2* mRNA products. Relative to the ortholog in *D. melanogaster* which also contains five CDSs, the protein sequence of Mipp2-PA in *D. simulans* has 98.23% matching identity with the *Mipp2* in *D. melanogaster* as determined by *blastp* ( Figure 1C ). Though this model is presented in the older GCA_000754195.3 assembly of *D. simulans* , inspection of the most recent RefSeq assembly of *D. simulans* ( GCF_016746395.2 ) also predicts the structure of the gene model in the orthologous genomic region that matches this annotation. The coordinates of the curated gene models can be found in NCBI at GenBank/BankIt using the accession BK063020 . These data are also available in Extended Data files below, which are archived in CaltechData.

## Methods

Detailed methods including algorithms, database versions, and citations for the complete annotation process can be found in Rele et al. (2023). Briefly, students use the GEP instance of the UCSC Genome Browser v.435 (https://gander.wustl.edu; Kent WJ et al., 2002; Navarro Gonzalez et al., 2021) to examine the genomic neighborhood of their reference IIS gene in the *D. melanogaster* genome assembly (Aug. 2014; BDGP Release 6 + ISO1 MT/dm6). Students then retrieve the protein sequence for the *D. melanogaster* reference gene for a given isoform and run it using *tblastn* against their target *Drosophila* species genome assembly on the NCBI BLAST server (https://blast.ncbi.nlm.nih.gov/Blast.cgi; Altschul et al., 1990) to identify potential orthologs. To validate the potential ortholog, students compare the local genomic neighborhood of their potential ortholog with the genomic neighborhood of their reference gene in *D. melanogaster* . This local synteny analysis includes at minimum the two upstream and downstream genes relative to their putative ortholog. They also explore other sets of genomic evidence using multiple alignment tracks in the Genome Browser, including BLAT alignments of RefSeq Genes, Spaln alignment of *D. melanogaster* proteins, multiple gene prediction tracks (e.g., GeMoMa, Geneid, Augustus), and modENCODE RNA-Seq from the target species. Detailed explanation of how these lines of genomic evidenced are leveraged by students in gene model development are described in Rele et al. (2023). Genomic structure information (e.g., CDSs, intron-exon number and boundaries, number of isoforms) for the *D. melanogaster* reference gene is retrieved through the Gene Record Finder (https://gander.wustl.edu/~wilson/dmelgenerecord/index.html; Rele et al *.,* 2023). Approximate splice sites within the target gene are determined using *tblastn* using the CDSs from the *D. melanogaste* r reference gene. Coordinates of CDSs are then refined by examining aligned modENCODE RNA-Seq data, and by applying paradigms of molecular biology such as identifying canonical splice site sequences and ensuring the maintenance of an open reading frame across hypothesized splice sites. Students then confirm the biological validity of their target gene model using the Gene Model Checker (https://gander.wustl.edu/~wilson/genechecker/index.html; Rele et al., 2023), which compares the structure and translated sequence from their hypothesized target gene model against the *D. melanogaster* reference gene model. At least two independent models for a gene are generated by students under mentorship of their faculty course instructors. Those models are then reconciled by a third independent researcher mentored by the project leaders to produce the final model. Note: comparison of 5' and 3' UTR sequence information is not included in this GEP CURE protocol (Gruys et al., 2025).

## Data Availability

Description: GFF, FASTA, and PEP file for model.. Resource Type: Model. DOI: https://doi.org/10.22002/j9c5e-k8q19
